# EXERCISE in pediatric autologous stem cell transplant patients: a randomized controlled trial protocol

**DOI:** 10.1186/1471-2407-12-401

**Published:** 2012-09-10

**Authors:** Carolina Chamorro-Viña, Gregory MT Guilcher, Faisal M Khan, Karen Mazil, Fiona Schulte, Amanda Wurz, Tanya Williamson, Raylene A Reimer, S Nicole Culos-Reed

**Affiliations:** 1 Faculty of Kinesiology, University of Calgary, 2500 University Drive NW, Calgary, AB T2N 1N4, Canada; 2 Section of Pediatric Oncology, Alberta Children’s Hospital, Calgary, Canada; 3 Faculty of Medicine, University of Calgary, Calgary, Canada; 4 Department of Psychosocial Resources, Tom Baker Cancer Centre, Calgary, Canada; 5 Department of Pathology & Laboratory, Faculty of Medicine, University of Calgary, Heritage Medical Research, 3300 Hospital Drive NW . Room 269, Calgary, Canada; 6 Division of Pediatric and Oncology, Alberta Children’s Hospital, 2888 Shaganappi Trail NW, Calgary, AB T3B 6A8, Canada; 7 Hematology, Oncology, Transplant Program, Alberta Children’s Hospital, 2888 Shaganappi Trail NW44, Calgary, AB T3B 6A8, Canada

**Keywords:** Pediatric, Hematopoietic stem cell transplant, Cancer, Exercise, Quality of life, Immune system, Physical activity levels

## Abstract

**Background:**

Hematopoietic stem cell transplantation is an intensive therapy used to improve survivorship and cure various oncologic diseases. However, this therapy is associated with high mortality rates and numerous negative side-effects. The recovery of the immune system is a special concern and plays a key role in the success of this treatment. In healthy populations it is known that exercise plays an important role in immune system regulation, but little is known about the role of exercise in the hematological and immunological recovery of children undergoing hematopoietic stem cell transplant. The primary objective of this randomized-controlled trial (RCT) is to study the effect of an exercise program (in- and outpatient) on immune cell recovery in patients undergoing an autologous stem cell transplantation. The secondary objective is to determine if an exercise intervention diminishes the usual deterioration in quality of life, physical fitness, and the acquisition of a sedentary lifestyle.

**Methods:**

This RCT has received approval from The Conjoint Health Research Ethics Board (CHREB) of the University of Calgary (Ethics ID # E-24476). Twenty-four participants treated for a malignancy with autologous stem cell transplant (5 to 18 years) in the Alberta Children’s Hospital will be randomly assigned to an exercise or control group. The exercise group will participate in a two-phase exercise intervention (in- and outpatient) from hospitalization until 10 weeks after discharge. The exercise program includes strength, flexibility and aerobic exercise. During the inpatient phase this program will be performed 5 times/week and will be supervised. The outpatient phase will combine a supervised session with two home-based exercise sessions with the use of the Wii device. The control group will follow the standard protocol without any specific exercise program. A range of outcomes, including quantitative and functional recovery of immune system, cytokine levels in serum, natural killer (NK) cells and their subset recovery and function, and gene expression of activating and inhibitory NK cell receptors, body composition, nutrition, quality of life, fatigue, health-related fitness assessment and physical activity levels will be examined, providing the most comprehensive assessment to date.

**Discussion:**

We expect to find improvements in immunological recovery and quality of life, and decreased acquisition of sedentary behavior and fitness deconditioning. The comprehensive outcomes generated in this RCT will provide preliminary data to conduct a multisite study that will generate stronger outcomes.

**Trial registration:**

Gov identification # NCT01666015

## Background

Hematopoietic stem cell transplantation (SCT) is an intensive therapy used in the treatment of various oncologic diseases [1]. This treatment has improved survivorship in recent years, but is associated with numerous negative physical and psychological side-effects as well as a spectrum of late effects [1-5]. Oeffinger et al. reported that one-third of childhood cancer survivors have severe or life-threatening medical complications 30 years after diagnosis, and those who received SCT compound the high-risk group [5]. Recent research attention has been focused on diminishing the impact of these potential negative side-effects and late-effects thereby, improving the quality of life (QOL) of pediatric cancer survivors.

There is increasing evidence that exercise (EX) is safe, beneficial and feasible in different stages of cancer treatment [3-6], including in immune-compromised patients [7]. However, scientific evidence suggests that children with cancer are more sedentary than their healthy peers [8,9]. It has been argued that childhood cancer survivors are subject to an insidious “spectrum of disuse” as a result of an overly cautious approach towards EX, fostered by concerned parents and environmental factors [10]. A potential explanation for this overprotective attitude may stem from a general lack of education about what a child can be expected to do after cancer treatments [11].

Huang et al. [3] in a recent review showed that the impact of EX on health and physical function in pediatric oncology is promising. However, only 15 published studies (1993-2011) have examined EX as an intervention [3]. Of these 15, only 4 were RCTs, and only 3 studied the effect of EX on immunological function [7,12,13]. Overall, these 3 studies found that EX was beneficial and did not impair immune system recovery in any way that could provoke concern for health. Furthermore, Chamorro et al. [7] showed that EX was safe in neutropenic patients, who were also able to avoid weight lost during hospitalization. This previous research is limited by small sample size and limited immunological analyses, including no assessment of NK cell function or cytokine environment. In addition, most of the previous EX interventions were in acute lymphoblastic leukemia patients undergoing maintenance therapy [3]. To our knowledge, only three English language publications described the effect of EX in children with cancer undergoing SCT [7,14,15]. These articles suggest that EX is safe, feasible and has a promising role in the recovery of children undergoing SCT.

Despite robust scientific evidence describing the positive effects of EX in the adult cancer population, limited research efforts have focused on exploring the translation of EX therapy to children. Specifically, the role of EX in the immune system recovery of pediatric patients undergoing SCT has not been examined sufficiently to date, however the results from the adult population [16-18] are promising. It is argued that improved QOL through an EX program is sufficient rationale to providing EX programming. However EX will be further supported if it can be shown to improve immune system recovery which may result in diminished risk of infection, hospitalization days and improve overall survival. This RCT will significantly add to the evidence on the effect of EX on the recovery of children undergoing SCT. A range of outcomes, including quantitative and functional recovery of immune system, e.g., different leukocyte cell subset counts in peripheral blood, cytokine levels in serum, NK cells and their subset recovery and function, and gene expression of activating and inhibitory NK cell receptors known as Killer Immunoglobulin like Receptors (KIR), body composition, nutrition, QOL, fatigue, health- related fitness assessment and physical activity levels will be examined, providing the most comprehensive assessment to date.

The purposes of the present study are:

### Primary

Study the effect of an EX program (in- and outpatient) on immune cell recovery in patients undergoing an autologous SCT.

### Secondary

Determine if an EX program performed in patients undergoing autologous SCT diminishes the deterioration of physical fitness, physical activity levels and improves QOL.

### Tertiary

Establish the program’s feasibility (uptake and adherence) to provide the impetus to run multi-centre research with larger populations in the future.

## Methods

### Study design

**Figure 1:**
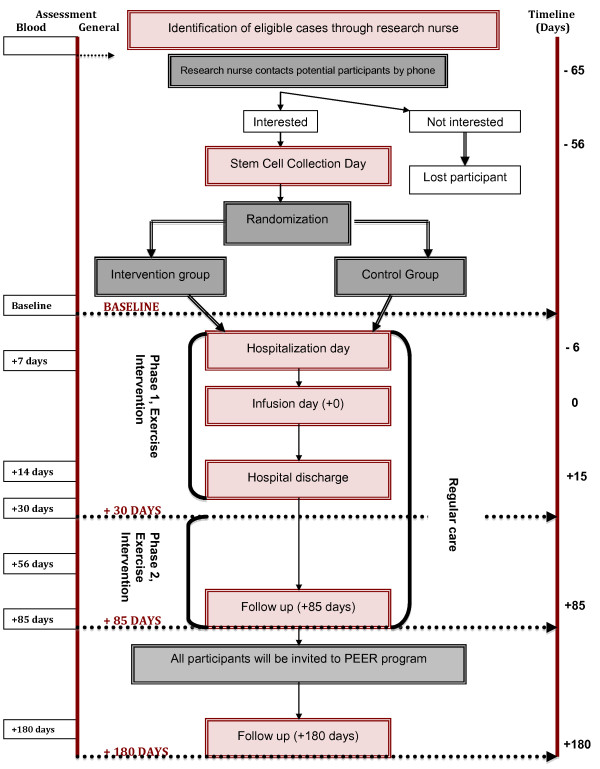
Participants flow through SCORE study.

SCORE “Stem Cell patients Ongoing Recovery through Exercise” is a RCT of pediatric cancer patients undergoing autologous SCT. Assessors will be blind to participant’s condition (EX or control) and all the statistical analyses will be performed maintaining blindness to condition. Blinding of assessors and statistical analyses ensures limited bias on outcomes, especially with subjective outcomes of interest. Flow through the study is depicted in Figure 1.

**Figure 2:**
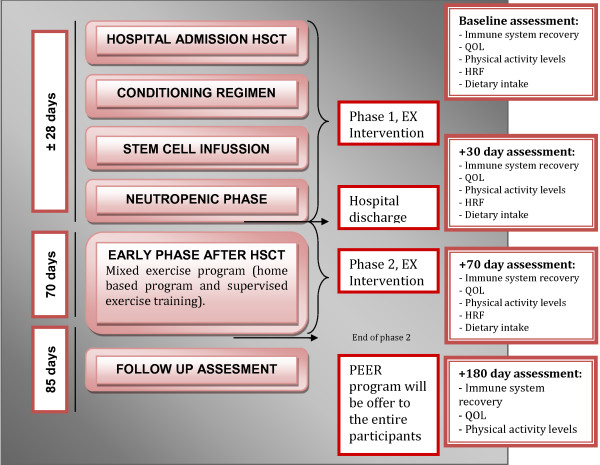
SCORE study protocol.

This study will examine the effect of two phases of an EX program on several health related outcomes in pediatric patients undergoing autologous SCT (Figure 2). The first phase of the EX program will be an inpatient intervention, beginning when the child is hospitalized undergoing conditioning therapy and continuing until discharge.

The second phase will be a 10-week outpatient intervention, beginning once the child is discharged. We will utilize a mixed EX program, including supervised (at the University of Calgary) and home-based training incorporating the use of the Nintendo ® Wii device™ (Wii Fit, Wii Dance and Sports™ games).

Assessments include measures of QOL, fatigue, health-related fitness (HRF), physical activity levels and hematological and immunological reconstitution. Assessments will be completed at baseline, 30, 85 and 180 days post infusion day (day 0+). Hematological and immunological reconstitution will be assessed at the time points described above, plus at 7, 15 and 56 days post reinfusion. The baseline assessment will be completed within the two-month period prior to the conditioning regimen. The 30 day assessment is intended to capture the effect of Phase 1, the EX program during the hospitalization period (from conditioning regimen up to discharge). The 85 day assessment is intended to capture the early survivorship recovery period after an autologous SCT and the effect of Phase 2, EX program during the outpatient phase. The 180 day assessment will provide a mid-term follow-up of physical activity, QOL, fatigue and hematological and immunological reconstitution. These outcomes will allow us to examine the impact of an EX program implemented early during the SCT process 3 months after finishing the EX intervention on (i) physical activity levels (ii) QOL and fatigue iii) hematological and immunological recovery

### Study population

A total of 24 children will be recruited for this RCT; 12 patients will be assigned to an EX training program and 12 subjects will be assigned to a control group (without any specific training). A sequence to allocate participants randomly will be generated by the software Research Randomizer, a research tool provided by the Social Psychology Network (http://www.socialpsychology.org). Allocation concealment will be ensured. After baseline assessments each participant will choose a sealed, non transparent envelop, which contains a number that will allocate the subjects to the control or EX group. The research coordinator will be responsible for the random allocation.

Eligibility criteria includes: (1) autologous SCT at Alberta Children’s Hospital (ACH) for malignancy, (2) age 5 to 18 years of age, (3) will be receiving myeloablative conditioning regimen, (4) no evidence of cardiac or pulmonary failure associated with treatment (shortening fraction (SF) ≥28%, ejection fraction (EF) ≥ 50%), (5) no functional nor cognitive limitation that would prohibit performance of the home-based training, (6) approval by treating oncologist for participants, (7) a parent or legal guardian must sign the consent form, and (8) children should express verbal assent to participate. The entire team of assessors and the persons performing the statistical analyses will be blinded to group assignment.

### Recruitment

Children undergoing autologous SCT will be recruited through the Section of Pediatric Oncology and Blood and Marrow Transplantation at ACH. ACH is the SCT centre for Alberta. The referring oncologist will identify potential subjects and the research nurse will contact them to present the study at least 4 weeks before the hospitalization to start autologous SCT treatment. Those subjects who are interested in the study will be asked to sign the consent form by the research nurse and will be contacted by the research assistant to set up the baseline assessment.

This on-site patient recruitment method will be more likely to enhance recruitment efforts than a mail out or other off-site method. Also, prospective subjects might feel more supported if the physician supports the research protocol. In order to obtain a homogenous sample, is known that the recruitment phase will take between 2-3 years to reach the sample described.

### Intervention and control group

#### Intervention group

Children in the intervention group will participate in an inpatient and outpatient mixed EX program including both resistance and aerobic training.

#### Control group

These subjects will not participate in any scheduled EX and will perform the same battery of tests as the intervention group. They will be offered an EX program upon completion of phase 2, to be held in the Thrive Centre, University of Calgary.

### Medical treatment – transplant protocol

High dose chemotherapy and autologous SCT will be performed for a given child as per the recommended therapy for a given disease and stage risk group. Only children receiving myeloablative conditioning will be eligible. Most children will have a history of Sarcoma, Lymphoma, Neuroblastoma or Germ cell tumor. Supportive care will be as per institutional practice.

### Sample size

Power calculations were derived using GPower 3.1 [19] The sample size/power calculation was based on relevant intervention literature [12,20,21] with natural killer cell activity as the primary outcome measure (Cohen’s d = .79). With an α of .05 and power of .80, a minimum sample size of 20 participants is required. To account for potential drop out, we will recruit an additional 20%, for a total of 24 participants.

### Data analysis

The raw data analysis will be performed using SPSS (v. 20.0) and will include descriptive, correlations, and repeated measures analysis of variance (ANOVA). Specifically, for the primary and second objectives, correlations will be run to examine the degree of relationship between the variables, and planned doubly multivariate repeated measures ANOVA’s will be performed to examine group differences between the intervention and control group. Descriptive statistics will be conducted for demographics, medical variables, adherence to the program, and physical activity behavior using means, standard deviations, and ranges as appropriate. As was previously mentioned, the statistician will be blinded to participant condition.

### EX intervention 

This RCT will be a biphasic EX intervention.

Phase 1 – The inpatient phase will be performed at the ACH during conditioning and the isolated phase of autologous SCT.

Phase 2 – The outpatient phase will take place after discharge. The participants will participate in a 10 week mixed supervised and home-based EX program utilizing the Wii device.

In both phases participants will wear a portable heart rate monitor (RS300X Polar) when at the EX sessions to ensure safety and target the aerobic EX intensity between 50% and 70% of heart rate reserve (HRR), depending on the physical status of each child.

The training session will be cancelled if the child has platelet levels below 10.000/ μl, hemoglobin levels below 8 g/dl, fever greater than 38°C, pain, diarrhea, hemorrhage or any other complication(s) the oncologist thinks may worsen with EX. Additionally, a session may be cancelled if the child doesn’t want to participate.

#### Phase One: In-Patient Exercise Program

This program will consist of combined aerobic (20-30 minutes) and resistance training, designed on the basis of our previous experience with pediatric patients with cancer, and following the rules of the institutions for global benchmarks for strength training and aerobic EX in children and adolescents [7,14,22]. During the neutropenic phase, all training equipment (including cycle ergometers) will be sterilized before each training session, and the instructors will wear appropriate medical attire.

The strength EXs, will engage the major muscle groups (arm curl, elbow extension, bench press, leg extension, half squat, abdominals, supine bridge, and rowing), performing one set of 12–15 repetitions per EX.

#### Phase Two: Outpatient Exercise Program

Following the in-patient EX program, the participants of the intervention group will engage in a 10-week EX program consisting of 3 weekly sessions. The participants from Calgary will perform a combined supervised and unsupervised/home-based exercise program (2 unsupervised and 1 supervised sessions/week) with an average duration of 60 minutes/session. The participants from outside of Calgary will perform only the home-based EX program 3 days/week. The supervised and unsupervised EX plans will be individualized based on fitness assessment outcomes and current health status.

##### Supervised EX program

The supervised training session (Calgary-based participants) will be performed at the Thrive Centre at the University of Calgary 1 time/week and will be similar to the inpatient intervention.

##### Home-Based Exercise Program

Designed by exercise professionals and supervised by the parents, it will include 20-30 minutes of aerobic EXs and 30 minutes of strength and stretching EXs using Nintendo’s Wii console and Wii Fit/Wii Sports/Wii Dance games (from now on, named as Wii Fit/Sports/Dance).

Parents and children are going to be instructed on how to control the intensity of the EXs. Training adherence will be reported in a logbook, including reported training days and reasons for missed EX sessions. Training sessions will be declared valid if 80% of the planned session were performed. Participants will be reminded and encouraged to engage in their home-based workouts when they come to the Thrive Centre, and all participants (including those residing outside of Calgary) will receive a weekly phone call (from the research assistant) to encourage the participants to continue performing the EX program and answer any questions they or their parents may have.

### Measures

Once consent has been obtained, the research nurse will be responsible for instructing participants (parents and pediatric patient) (1) on the use of the accelerometers and log book, (2) on how to perform the food record, and (3) on the QOL assessment. The research coordinator will be responsible for contacting participants to perform the baseline HRF assessment. All baseline assessments will be performed before any conditioning regimen is administered. Additionally, the research coordinator will ensure all assessments are administered according to the protocol.

We will perform: (1) Hematological and Immunological Recovery, (2) Health and Lifestyle Assessment and (3) Health-Related Fitness Assessments (HRF).

### Hematological and immunological recovery

#### Specimen collection

At baseline 2 Buccal Swabs will be collected to use for deoxyribonucleic acid (DNA) extraction. DNA specimens will be used to perform genotyping of activating and inhibitory KIR genes. The patient should not eat or drink anything for one hour prior to the specimens being taken. We will take one swab and rub the inside of right and left patient’s cheek for approximately 10 seconds. The swab will air dry for 1-2 minutes prior to replacing it into its container.

The baseline blood draw will be performed at the Unit 1 in the ACH (within 7 days prior to conditioning and transplant). The maximum blood volume to be drawn will be determined based on patient weight and other ordered blood work. A minimum of 7 mL will be required. The blood sample collection will be performed at baseline, day 7, 14, 30, 56, 85 and 180.

To avoid any acute EX effects, we will discourage any specific EX training 24 h before blood draws in the samples that will be collected at baseline and after transplant on day 14, 30, 56, 85 and 180. Research samples may be collected via central line or peripheral draw. For this study, we will store 1 aliquot each for peripheral blood mononuclear cells (PBMNCs) for every blood specimen serum. The aliquoted serum samples will be stored in our -86°C freezers and PBMNCs will be cryo-preserved at -150°C using liquid nitrogen based cell storage facility. All immune functional assays will be performed on cry preserved PBMNCs.

#### Hematological and immune system assessment

Assessment of the impact of the EX intervention on the immune recovery of pediatric autologous SCT patients will be based on four parameters (a) Recovery of different leukocyte cell subset (e.g., T cells, NK cells, B cells, monocytes, neutrophils etc) in peripheral blood, (b) Expression of activating and inhibitory KIRs (c) Function of NK cells and their subsets and (d) Levels of different pro-inflammatory and regulatory cytokines in serum,

a) Recovery of NK cells and other immune cells subsets will be performed by flow cytometry enumeration of different blood leukocyte subsets including NK cells and its subsets (cytolysis and regulatory NK cells), T cells and its subsets (CD4, CD8, regulatory, naive and memory T cells), B cells, monocytes, neutrophils and dendritic cells.

b) Gene/allele carriage analysis for 8 inhibitory KIR genes (2DL1, 2DL2, 2DL3, 2DL4, 2DL5, 3DL1, 3DL2 and 3DL3); 6 activating KIR genes (3DS1, 2DS1, 2DS2, 2DS3, 2DS4 and 2DS5) and two pseudo genes (2DP1 and 3DP1) will be performed for all 24 patients. Gene expression analysis of 14 KIR genes will be done by RNA based real-time PCR analysis. Total RNA will be extracted from the PBMNCs for each time point and cDNA will be prepared using random primers and M-MLV reverse transcriptase enzyme. A quantitative real time PCR analysis will be performed to assess the expression of genes encoding for 8 inhibitory and 6 activating KIR genes. Quantitative PCR will be carried out with SYBR Green PCR method. The mRNA expression of different KIR genes will be normalized against GAPDH, and fold change in expression will be determined using ddCt method. Additionally, a flow-cytometry based protein expression analysis for 7 KIRs (4 activating and 3 inhibitory KIRs) on NK cells will also be performed. PBMNCs from each subject for each time point will be stained with fluorochrome labeled antibodies against different KIR molecules and markers specific for NK cells. Cells will be analyzed by multi-color flow cytometry. To quantify the amount of KIR expression on NK cells, we will determine the mean fluorescence intensity (MFI) for each KIR on NK cells.

c) NK function will be assessed by measuring in vitro cytokine secretion and degranulation by different NK cell subsets (cytolysis and regulatory NK cells) on incubation with K-562 cell lines. PMNCs will be cultured with and without K562 cell in RPMI supplemented with 5% fetal bovine serum, glutamine (2 mM), Penicillin (100 U/ml) and Streptomycin (0.1 mg/ml) at 37°C in a humidified atmosphere containing 5% CO_2_. To measure degranulation, fluorochrome-conjugated antibody against human CD107a (a surrogate marker of degranulation) will be added to the cells at the start of culture as CD107a is only transiently expressed on T cell surface following activation-induced degranulation and it becomes internalized over time. Monessen will also added to the culture from beginning to ensure blockade of cytokine export from cells to the extra-cellular space and to provide neutral pH that maintains the fluorescence of FITC-conjugated CD107a antibody bound to CD107a, when internalized into the cell. At the end of the culture, cells will be stained with fluorochrome labeled antibodies against CD, CD3, CD56, CD16 and IFNγ and will be analyzed by multi-color flow cytometry. A cytolysis NK cell will be defined as CD3^neg^ CD56^dim^ CD16^pos^ NK cell while a regulatory NK cell will be defined as CD3^neg^ CD56^dim^ CD16^pos^ IFNγ^pos^ NK cell. Positivity for IFNγ and CD107a will correspond to the cytokine producing and degranulation functions respectively.

d) Assessment of cytokine levels in serum: A total of 30 cytokines including TNF-α, IFN-γ, IL-10, IL-6, IL-1α, IL-1β, IL-1R, IL-2, IL-12, TGF-β IL-15, and IL-17 etc will measured in the serum specimens for each time point using a bead-based array on Luminex.

### Health and lifestyle assessment

#### Baseline health

Baseline demographic and health characteristics of the study participants will include demographic characteristics, health record information (type of cancer, disease status, co-morbid conditions, cancer treatment received, side-effects of treatment) and information related to SCT (platelet and neutrophil engraftment, conditioning regimen received, toxicities due to conditioning regimen, documented infection, incidence of fever). These questionnaires will be filled out by the physician at baseline, 15, 30, 60, 85 and 180 days after stem cell infusion.

#### Dietary habits

Usual dietary intake will be assessed at four time points (baseline, 30, 85 and 180 days after infusion) using a 3-day dietary record [23]. Dietary records will be analyzed by a Registered Dietitian using Diet Analysis Plus 10.0 software (Thomson Wadsworth, Toronto, Canada). Participants and their parents will be instructed as to how to fill out these records by the research nurse.

#### Quality of life

Quality of life will be assessed using the Pediatric quality of life inventory (PedsQL) general and cancer module as a self-report, and PedsQL and Behavior Assessment System for Children (BASC-2) as parent proxy report. The PedsQL Measurement Model is a modular approach to measuring QOL in healthy children and adolescents and those with acute and chronic health conditions. The PedsQL Measurement Model integrates seamlessly both generic core scales and disease-specific modules into one measurement system. The 23-item PedsQL Generic Core Scales were designed to measure the core dimensions of health as delineated by the World Health Organization, including physical, emotional, social and school functioning. In addition, the cancer module will be assessed to provide greater measurement sensitivity. The reliability and validity of the PedsQL general and cancer module has been shown in pediatric cancer populations [24]. Participants will complete this measure.

The BASC-2 [25] is a standardized report measure of child behavior consisting of 150 items with self, parent proxy, or teacher report forms. The BASC yields scores for externalizing (i.e., hyperactivity, aggression, conduct problems) and internalizing (i.e. anxiety, depression, and summarization) behavior problems as well as for adaptive skills. Items are rated using a 4-point ordinal scale. Raw scores are converted into T scores. T-scores of 60 or greater are indicative of clinically significant behavior problems. Parents will be asked to complete this measure as a means of enhancing QOL measurement sensitivity.

#### Fatigue

Fatigue will be assessed using the multidimensional PedsQL fatigue scale. The recently developed 18-item PedsQL Multidimensional Fatigue Scale was designed to measure fatigue in pediatric patients and comprises the General Fatigue Scale (6 items), Sleep/Rest Fatigue Scale (6 items), and Cognitive Fatigue Scale (6 items). Reliability and validity of the pediatric multidimensional fatigue scale has been shown in cancer population [24]. Participants will complete this measure.

#### Objective measurements of physical activity

A triaxial accelerometer (Actual – Phillips respironics) will be used as an objective assessment of physical activity levels. Actual measures and records time-stamped acceleration in all directions, providing an index of physical activity intensity. The digitized values are summed over a user-specified interval of 1 min, resulting in a count value per minute (counts per minute). Accelerometer signals are also recorded as steps per minute. This gold standard measure provides a reliable and objective measure of physical activity levels, previously validated and tested for reliability [26,27].

The activity monitor (AM) will be put on when the participant gets up from bed each day and worn until the participant is ready to fall asleep. Participants can go about their normal, daily activities, including rigorous EX, swimming or bathing. Participants will be instructed to wear the activity monitor over their right hip on an elasticized belt for seven consecutive days. The AM can be worn over or under clothing. Additionally, participants will be asked to record, in a daily log, the time they put on and took off the monitor each day. This protocol and device was recently used in a study to asses physical activity among healthy Canadian children between 6-19 yr [28]. The research nurse will explain the instructions about how to use the AM and daily log at their first meeting.

### Health-related fitness assessments

The HRF assessments will be performed by a certified exercise physiologist. Participants will complete the assessments in the following order: resting blood pressure (BP), heart rate (HR) and electrocardiogram; body composition; musculoskeletal fitness (flexibility, muscular strength and endurance); functional mobility test and cardiorespiratory fitness. Adequate recovery and hydration will be provided, with the total fitness assessment time approximately 2.0 hours.

#### Submaximal aerobic test

Resting HR will be measured using a heart rate monitor (Polar Electro RS300X, Finland) and resting BP (mmHg) will be measured following the Fourth Report published by the U.S. Department of Health and Human Services [29]. All participants will perform a submaximal EX test on a treadmill. A modified protocol suggested by Bar-Or (1993) adjusted by height will be used. The initial velocity of the test will be determined upon child height (Table 1) based on Bar-Or suggestion for healthy kids maximal testing [30]. All participants will start the test with an upgrade of 5%. Every 2 minutes (Tanner stages I-IV) and every 3 minutes (Tanner stage V), the slopes will increase 2.5% until 80% of age-predicted maximal HR is achieved. Age-predicted maximal HR will be calculated using the equation 208- (0.7× age) [31].

**Table 1:** Sub maximal aerobic test; setting speed according to height of each participant

**STATURE(cm)**	**SPEED (km/h)**
≤ 109.9 cm	3 km/h
110–129.9 cm	4 km/h
≥ 130 cm	4.8 km/h

All the children will be verbally encouraged during the test. Gas-exchange data will be measured in a mixed metabolic cart (True One Metabolic Cart, ParVo Medics Inc, Utah, U.S.) and specific pediatric face-masks (Hans Rudolph Model 8930, 8940, 8950, 8960). The duration of the test, the maximal load reached (velocity and slope), and the maximal distance walked will be recorded. In addition, parameters including ventilation (VE), ventilatory equivalent of carbon dioxide (VCO_2_), respiratory exchange ratio (RER), and Respiratory Quotient (RQ) will be recorded.

Oxygen uptake and HR will be determined at Ventilatory threshold (VT). VT will be determined using the criteria of an increase in both the ventilatory equivalent of oxygen (VE . VCO_2_^-1^) and end-tidal pressure of oxygen (PetO_2_) with no increase in the VCO_2_ while RER remained below 1.0 [32]. All the EX tests will be performed under similar environmental conditions and at the same time of the day (10:00 am – 13:00 pm). Participants will be directed to consume their usual breakfast 3 hours prior to the testing.

Previous familiarization sessions will not be possible due to the nature of the study population (i.e., approximately ½ of participants will be from outside of Calgary). To diminish the anxiety and fear that fitness tests may generate, participants and parents will have the opportunity to watch a video explaining and showing all assessments.

#### Functional mobility test

This test is used to measure the capacity of the children to perform daily activities. We will perform the timed up and go test (TUG) in 3 m [33,34]. For the TUG, the child is sitting with 90° hip and knee flexion and at a given signal the child will get up, walk 3 m, turn and come back as fast as possible without running. The researcher measures the time spent performing this test. The test will be performed three times and the best time will be registered. Between each attempt the child will rest for 2 minutes.

#### Musculoskeletal fitness

##### Muscular endurance

Muscular endurance is described as the ability of a muscle or a muscle group, to generate force repeatedly or for an extended period of time. Muscular endurance can be determined by measuring the number of repetitions or time to fatigue. We are going to ask the children to perform three tests: (a) partial curl-up, (b) modified push-up and (c) sit and stand test.

a) Partial curl-up will assess the muscular endurance of the abdominal muscles. The FITNESSGRAM protocol will be used [35]

b) Modified push-up: This test measures the endurance of upper body muscles.

The modified push-up will require the participant to start with hands shoulder-width apart with the elbows and body straight. The knees will be flexed 90° and the ankles crossed. The test consists of doing as many repetitions as possible continuously until failure. To ensure the participant is properly completing the push up, they will have to lower their torso until they come into contact with a foam roller (10 cm diameter) on the ground (rather than a fist).

c) Squat test: Will assess the muscular endurance of the lower body. The participant stands in front of a chair, facing away from it, with their feet shoulder width apart. The participant squats down lightly touching the chair (located at 90 ˚ hip flexion) with their backside before standing back up and repeats this sequence of movements for 30 seconds [36]. The assistant counts and records the number of successfully completed squats.

##### Strength assessment

Strength is defined as the maximal force or torque developed by a muscle, or a muscle group. Maximum isometric contraction values will be done by assessing (a) knee extension and (b) grip strength.

a) Knee extension will be performed by using the “break” technique, in which the examiner gradually overcomes the muscle force and stops at the moment the extremity gives away [37,38]. All tests will be performed with the tested limb segment in a position that will be not affected by gravity. The tester will manually stabilize the body part proximal to the tested limb segment during testing. Before the test, the assessor will demonstrate to the patient the muscle contraction. The piston of the dynamometer will be held perpendicular to the anterior surface of leg. The plate of the dynamometer will be placed just proximal to the ankle on the anterior surface of the leg. The subject will be seated on the edge of a padded mat table with the knee flexed 90°. Measures of strength of the right and left sides will be assessed. A sum will be determined in kilograms from the best score of 2 trials recorded for each muscle group.

b) Grip strength will also be assessed using a hand-dynamometer (Baseline®). Measures of strength of the right and left sides will be assessed. A sum will be determined in kilograms from the best score of 2 trials recorded for each muscle group, according to the CPAFLA protocol [39].

#### Flexibility

Sit and reach test measures the flexibility of the hamstrings and lower back muscles [39]. CPAFLA protocol will be followed [39].

#### Body composition

Standing height and body mass will be measured. We will measure standing height to the nearest 0.1 cm with a clinical stadiometer (SECA, Birmingham, UK), while children stand barefoot. Body mass will be measured to the nearest 0.01 kg using a balance scale (Health o Meter, Illinois, US) with the subjects in their underwear and will calculate body mass index (BMI) as weight height [mass (kg)/height (m^2^)]. Estimated children’s fat-free mass (FFM) will be calculated from the following equation (1): FFM = body mass – fat mass (FM), where FM = 4.95 x Body density (BD) x body mass. Children’s BD will be estimated from skin fold thickness at the triceps, biceps, sub scapular, and suprailiac area. In all subjects, each skin fold will be obtained in duplicate by the same researcher using standard equipment (Harpender skin fold caliper, Bath international, UK) and the mean value per skin fold utilized for statistical analysis [39]. A third skin fold measure will be taken if the difference between the first two measures is greater than 4 mm. Different equations depending on children’s age and gender will be used to calculated BD (1) BD = 1,169 - 0,0788 * log Σ 4 skin folds and BD = 1,2063 - 0,0999 * log Σ 4 skin folds (for boys and girls, respectively, aged ≤12 yr) and (2) BD = 1,1533 - 0,0643 * log Σ 4 skin folds and BD = 1,1369 - 0,0598 * log Σ 4 skin folds (for boys and girls, respectively, aged ≥ 13 yr) [40].

## Discussion

The primary focus of the SCORE study will be to identify the effect of an early EX intervention in the immune system recovery of pediatric patients undergoing autologous SCT. Other important health outcomes will include the intervention impact on QOL and fatigue. We will also be able to examine the mediators and moderators of any observed associations between PA, HRF, and health outcomes after SCT at a mid-term assessment point. Finally, we will be able to identify if an early intervention will be able to diminish the acquisition of a sedentary lifestyle,

Taken together, these data will provide a detailed and complete understanding of the effect of an EX program in pediatric cancer patients undergoing autologous SCT. From our knowledge, this study will be one of the most comprehensive studies in this population, and will provide an initial database along with the impetus to promote a multisite study. A multisite study is the ideal approach to examine the impact of EX on immunological and patient-reported outcomes in the pediatric oncology population, due to relatively small sample sizes at any one centre.

The SCORE study is designed to address the following research themes through pilot data:

1. Hematological and immunological reconstitution and exercise. The primary aim of this project is to examine the associations between EX, recovery of hematological and immunological reconstitution, and the relation with some disease outcomes in pediatric cancer patients undergoing autologous SCT (including duration of neutropenia, thrombocytopenia, anemia, fever, toxicity degree, chemotherapy completion rate infectious complications and event free survival) [41]. The clinical utility of autologous SCT is limited in part by treatment related toxicity [42]. Also, an absolute lymphocyte recovery of 500 cells/μl or more at day 15 after autologous SCT has been reported as a powerful and independent prognostic indicator of clinical outcomes. NK cells have antitumor activity, therefore an early NK cell engraftment with an early anti-tumor immune-surveillance might have a direct impact on survival post autologous SCT. Preliminary studies in children with cancer showed a decrease in NK lytic activity before and after cessation of cancer chemotherapy, especially in those with progressive disease [43,44]. Physical activity has the ability to promote an immune modulation that may involve multiple biological pathways, including a reduction in inflammation, an enhancement of anti-tumor response and may modulate killer cell immunoglobulin-like receptors (KIRs) [45]. Neither of the aforementioned mechanism has been studied in detail [46]. More research is needed to determine which inflammatory mediators and antitumor immune mechanism are more sensitive to EX.

2. Exercise, health-related fitness, quality of life and fatigue questionnaires. The specific objectives of this study are to examine the effect of an EX program on (a) QOL and fatigue outcomes across the SCT process and immediately after discharge, (b) to examine the associations between HRF indices, physical activity and QOL and fatigue outcomes.

Pediatric cancer patients undergoing SCT have an elevated risk for poor QOL and fatigue both during treatment and into survivorship, however this issue has to be studied in depth [4]. Some evidence suggests that EX may be a beneficial tool to improve fatigue and QOL in children with cancer [47]. New research suggest that fatigue is strongly associated with a diminish in physical activity levels [48], thus it is imperative to break the vicious cycle of fatigue in this population.

Systematic reviews support the promising role of EX as a safe and effective intervention to improve HRF and quality of life and fatigue outcomes in children with cancer [3,4]. However, most of the research was performed in children with ALL. Only three articles have been published showing the effect of an EX intervention in children undergoing SCT and the outcomes are promising including improve in cardio respiratory capacity, strength, fatigue and QOL [7,14,15]. This study will provide details about the effect of an EX program on fatigue and QOL as a self-report outcome and also will be compared with parent’s proxy report.

3. The effects of an EX intervention on body composition

The aim of this study is to determine if an early EX intervention during SCT will (a) avoid loss of body weight. Survivors of pediatric patients who went undergoing a SCT are at an increased risk to have less lean mass and an excess of fat mass compared with healthy peers [49].

Given the scarcity of literature on the effect of an EX program on the immune system recovery, QOL, physical activity levels and HRF measures for pediatric cancer patients undergoing SCT, the SCORE study will generate new knowledge and be instrumental in developing future clinical and community-based programming for this population. This will be the largest known study in children undergoing autologous SCT and will provide us with valuable information for a future multisite study. This research could add biologic plausibility to the association between EX and immune system recovery after autologous SCT that may identify new targets for interventions, and inform clinical recommendations for improving survival. Ultimately, this project will enable us to achieve more effective targeted interventions that help pediatric cancer patients achieve healthy levels of PA and HRF and with the hope of optimizing disease and treatment related outcomes.

## Abbreviations

SCORE, (Stem Cell patients Ongoing Recovery through Exercise); SCT, Hematopoietic stem cell transplantation; QOL, Quality of life; EX, Exercise; RCT, Randomized controlled trial; NK, Natural Killer; KIR, Killer immunoglobulin like receptor; HRF, Health-related fitness; PBMNC, Peripheral blood mononuclear cell; DNA, Deoxyribonucleic acid; MFI, Mean fluorescence intensity; PedsQL, Pediatric quality of life inventory; BASC-2, Behavior assessment system for children; AM, Activity monitor; BP, Blood pressure; HR, Heart rate; VE, Ventilation; VCO2, Ventilatory equivalent of carbon dioxide; VT, Ventilatory threshold; RER, Respiratory exchange ratio; RQ, Respiratory quotient; PetO2, End tidal pressure of oxygen.

## Competing interests

The entire authors listed in this manuscript declare that they have no competing interests.

## Authors’ contributions

CC-V conceived the study and participated in its design and coordination as well as helped draft the manuscript. GG participated in the design of the study, facilitated the in-hospital setting and helped to draft the manuscript. FK was responsible for choosing the methods to carry out the immune system assessment. Additionally he will carry out the immunoassays, data analysis of immune system assessment and he helped to draft the manuscript. KM as a research nurse who will help us in the recruitment of the participants and ensure the entire participant will have done the blood draw properly. She helped to draft the manuscript. FS will participate in the analysis of the quality of life and fatigue questionnaires. AW will participate in the development of the exercise intervention and will help coordinate testing times. She also helped to draft the manuscript. TW will be responsible to perform and interpret the health-related fitness assessments. RR will be responsible for analyzing the 3 day dietary intake records. NCR as a PI will be responsible to oversee the study and data analysis and all subsequent manuscripts and dissemination of the work. She also conceived and participated in the design and coordination of the study. All authors read and approved the final manuscript.

## Pre-publication history

The pre-publication history for this paper can be accessed here:

http://www.biomedcentral.com/1471-2407/12/401/prepub

## References

[B1] Miano M Labopin M Hartmann O Angelucci E Cornish J Gluckman E Locatelli F Fischer A Egeler RM Or R Haematopoietic stem cell transplantation trends in children over the last three decades: a survey by the paediatric diseases working party of the European Group for Blood and Marrow Transplantation Bone Marrow Transplant 2007 39 2 89 99 17213848 10.1038/sj.bmt.1705550

[B2] Oeffinger KC Hudson MM Landier W Survivorship: childhood cancer survivors Primary care 2009 36 4 743 780 10.1016/j.pop.2009.07.007 19913185

[B3] Huang TT Ness KK Exercise interventions in children with cancer: a review International journal of pediatrics 2011 2011 461512 22121378 10.1155/2011/461512 PMC3205744

[B4] San Juan AF Wolin K Lucia A Physical activity and pediatric cancer survivorship Recent results in cancer research Fortschritte der Krebsforschung Progres dans les recherches sur le cancer 2011 186 319 347 21113771 10.1007/978-3-642-04231-7_14

[B5] Oeffinger KC Mertens AC Sklar CA Kawashima T Hudson MM Meadows AT Friedman DL Marina N Hobbie W Kadan-Lottick NS Chronic health conditions in adult survivors of childhood cancer N Engl J Med 2006 355 15 1572 1582 10.1056/NEJMsa060185 17035650

[B6] Oeffinger KC Robison LL Childhood cancer survivors, late effects, and a new model for understanding survivorship Jama 2007 297 24 2762 2764 10.1001/jama.297.24.2762 17595279

[B7] Chamorro-Vina C Ruiz JR Santana-Sosa E Gonzalez Vicent M Madero L Perez M Fleck SJ Perez A Ramirez M Lucia A Exercise during hematopoietic stem cell transplant hospitalization in children Med Sci Sports Exerc 2010 42 6 1045 1053 19997035 10.1249/MSS.0b013e3181c4dac1

[B8] Warner JT Bell W Webb DK Gregory JW Daily energy expenditure and physical activity in survivors of childhood malignancy Pediatr Res 1998 43 5 607 613 10.1203/00006450-199805000-00008 9585006

[B9] Reilly JJ Ventham JC Ralston JM Donaldson M Gibson B Reduced energy expenditure in preobese children treated for acute lymphoblastic leukemia Pediatr Res 1998 44 4 557 562 10.1203/00006450-199810000-00015 9773846

[B10] Aznar S Webster AL San Juan AF Chamorro-Vina C Mate-Munoz JL Moral S Perez M Garcia-Castro J Ramirez M Madero L Physical activity during treatment in children with leukemia: a pilot study Appl Physiol Nutr Metab 2006 31 4 407 413 10.1139/h06-014 16900230

[B11] Robertson AR Johnson DA Rehabilitation and development after childhood cancer: can the need for physical exercise be met? Pediatr Rehabil 2002 5 4 235 240 12745903 10.1080/1363849031000094072

[B12] Shore S Shepard RJ Immune responses to exercise in children treated for cancer J Sports Med Phys Fitness 1999 39 3 240 243 10573667

[B13] Ladha AB Courneya KS Bell GJ Field CJ Grundy P Effects of acute exercise on neutrophils in pediatric acute lymphoblastic leukemia survivors: a pilot study J Pediatr Hematol Oncol 2006 28 10 671 677 10.1097/01.mph.0000243644.20993.54 17023828

[B14] San Juan AF Chamorro-Vina C Moral S Fernandez Del Valle M Madero L Ramirez M Perez M Lucia A Benefits of intrahospital exercise training after pediatric bone marrow transplantation Int J Sports Med 2008 29 5 439 446 10.1055/s-2007-965571 17960520

[B15] Rosenhagen A Bernhorster M Vogt L Weiss B Senn A Arndt S Siegler K Jung M Bader P Banzer W Implementation of structured physical activity in the pediatric stem cell transplantation Klin Padiatr 2011 223 3 147 151 10.1055/s-0031-1271782 21462101

[B16] Peters C Lotzerich H Niemeier B Schule K Uhlenbruck G Influence of a moderate exercise training on natural killer cytotoxicity and personality traits in cancer patients Anticancer Res 1994 14 3A 1033 1036 8074446

[B17] Na YM Kim MY Kim YK Ha YR Yoon DS Exercise therapy effect on natural killer cell cytotoxic activity in stomach cancer patients after curative surgery Arch Phys Med Rehabil 2000 81 6 777 779 10857523 10.1016/s0003-9993(00)90110-2

[B18] Kim SD Kim HS Effects of a relaxation breathing exercise on anxiety, depression, and leukocyte in hemopoietic stem cell transplantation patients Cancer Nurs 2005 28 1 79 83 10.1097/00002820-200501000-00012 15681986

[B19] Faul F Erdfelder E Lang AG Buchner A G*Power 3: a flexible statistical power analysis program for the social, behavioral, and biomedical sciences Behavior research methods 2007 39 2 175 191 10.3758/BF03193146 17695343

[B20] Fairey AS Courneya KS Field CJ Mackey JR Physical exercise and immune system function in cancer survivors: a comprehensive review and future directions Cancer 2002 94 2 539 551 10.1002/cncr.10244 11900239

[B21] Fairey AS Courneya KS Field CJ Bell GJ Jones LW Mackey JR Randomized controlled trial of exercise and blood immune function in postmenopausal breast cancer survivors J Appl Physiol 2005 98 4 1534 1540 10.1152/japplphysiol.00566.2004 15772062

[B22] Faigenbaum AD Kraemer WJ Blimkie CJ Jeffreys I Micheli LJ Nitka M Rowland TW Youth resistance training: updated position statement paper from the national strength and conditioning association J Strength Cond Res 2009 23 5 Suppl S60 S79 19620931 10.1519/JSC.0b013e31819df407

[B23] Tremblay A Sévigny J Leblanc C Bouchard C The reproducibility of a three-day dietary record Nutr Res 1983 3 6 819 830 10.1016/S0271-5317(83)80035-9

[B24] Varni JW Burwinkle TM Katz ER Meeske K Dickinson P The PedsQL in pediatric cancer: reliability and validity of the Pediatric Quality of Life Inventory Generic Core Scales, Multidimensional Fatigue Scale, and Cancer Module Cancer 2002 94 7 2090 2106 10.1002/cncr.10428 11932914

[B25] Wolfe-Christensen C Mullins L Stinnett T Carpentier M Fedele D Use of the Behavioral Assessment System for Children 2nd Edition: Parent Report Scale in Pediatric Cancer Populations Journal of Clinical Psychology in Medical Settings 2009 16 4 322 330 10.1007/s10880-009-9174-7 19756977

[B26] Puyau MR Adolph AL Vohra FA Zakeri I Butte NF Prediction of activity energy expenditure using accelerometers in children Medicine and science in sports and exercise 2004 36 9 1625 1631 15354047

[B27] Pfeiffer KA McIver KL Dowda M Almeida MJ Pate RR Validation and calibration of the Actical accelerometer in preschool children Med Sci Sports Exerc 2006 38 1 152 157 10.1249/01.mss.0000183219.44127.e7 16394968

[B28] Colley RC Janssen I Tremblay MS Daily step target to measure adherence to physical activity guidelines in children Med Sci Sports Exerc 2012 44 5 977 982 10.1249/MSS.0b013e31823f23b1 22051570

[B29] The fourth report on the diagnosis, evaluation, and treatment of high blood pressure in children and adolescents Pediatrics 2004 114 2 Suppl 4th Report 555 576 15286277

[B30] Docherty D Measurment in pediatric exercise sciences 1996 Human Kinetics, Champaign, Illinois

[B31] Mahon AD Marjerrison AD Lee JD Woodruff ME Hanna LE Evaluating the prediction of maximal heart rate in children and adolescents Res Q Exerc Sport 2010 81 4 466 471 10.5641/027013610X13088600029337 21268470

[B32] Caiozzo VJ Davis JA Ellis JF Azus JL Vandagriff R Prietto CA McMaster WC A comparison of gas exchange indices used to detect the anaerobic threshold J Appl Physiol 1982 53 5 1184 1189 7174412 10.1152/jappl.1982.53.5.1184

[B33] Gocha Marchese V Chiarello LA Lange BJ Strength and functional mobility in children with acute lymphoblastic leukemia Med Pediatr Oncol 2003 40 4 230 232 10.1002/mpo.10266 12555250

[B34] San Juan AF Fleck SJ Chamorro-Vina C Mate-Munoz JL Moral S Garcia-Castro J Ramirez M Madero L Lucia A Early-phase adaptations to intrahospital training in strength and functional mobility of children with leukemia J Strength Cond Res 2007 21 1 173 177 10.1519/00124278-200702000-00031 17313277

[B35] The Cooper Institute Fitnessgram & Activitygram Test Administration Manual 2010 United States of America: Human Kinetics, Dallas, Texas

[B36] Jones CJ Rikli RE Beam WC A 30-s chair-stand test as a measure of lower body strength in community-residing older adults Res Q Exerc Sport 1999 70 2 113 119 10380242 10.1080/02701367.1999.10608028

[B37] Beenakker EA van der Hoeven JH Fock JM Maurits NM Reference values of maximum isometric muscle force obtained in 270 children aged 4-16 years by hand-held dynamometry Neuromuscular disorders : NMD 2001 11 5 441 446 10.1016/S0960-8966(01)00193-6 11404114

[B38] Ness KK Morris EB Nolan VG Howell CR Gilchrist LS Stovall M Cox CL Klosky JL Gajjar A Neglia JP Physical performance limitations among adult survivors of childhood brain tumors Cancer 2010 116 12 3034 3044 10.1002/cncr.25051 20564409 PMC3554250

[B39] CSEP-Health & Fitness Program’s Health-Related Appraisal and Counselling Strategy The Canadian Physical Activity 2003 Third edition © 2003 Fitness & Lifestye Approach Protocol (CPAFLA), Ottawa, Ontario

[B40] Durnin JV Rahaman MM The assessment of the amount of fat in the human body from measurements of skinfold thickness Br J Nutr 1967 21 3 681 689 10.1079/BJN19670070 6052883

[B41] Courneya KS Segal RJ Mackey JR Gelmon K Reid RD Friedenreich CM Ladha AB Proulx C Vallance JK Lane K Effects of aerobic and resistance exercise in breast cancer patients receiving adjuvant chemotherapy: a multicenter randomized controlled trial J Clin Oncol 2007 25 28 4396 4404 10.1200/JCO.2006.08.2024 17785708

[B42] Kalwak K Gorczynska E Toporski J Turkiewicz D Slociak M Ussowicz M Latos-Grazynska E Krol M Boguslawska-Jaworska J Chybicka A Immune reconstitution after haematopoietic cell transplantation in children: immunophenotype analysis with regard to factors affecting the speed of recovery Br J Haematol 2002 118 1 74 89 10.1046/j.1365-2141.2002.03560.x 12100130

[B43] Gallego-Melcon S Espanol Boren T Sanchez De Toledo J Prats Vinas J Natural killer cell function in children with malignant solid neoplasias Med Pediatr Oncol 1991 19 3 175 181 10.1002/mpo.2950190306 2023566

[B44] Alanko S Salmi TT Pelliniemi TT Recovery of natural killer cells after chemotherapy for childhood acute lymphoblastic leukemia and solid tumors Med Pediatr Oncol 1995 24 6 373 378 10.1002/mpo.2950240607 7715543

[B45] Maltseva DV Sakharov DA Tonevitsky EA Northoff H Tonevitsky AG Killer cell immunoglobulin-like receptors and exercise Exerc Immunol Rev 2011 17 150 163 21446357

[B46] Walsh NP Gleeson M Shephard RJ Woods JA Bishop NC Fleshner M Green C Pedersen BK Hoffman-Goetz L Rogers CJ Position statement. Part one: Immune function and exercise Exerc Immunol Rev 2011 17 6 63 21446352

[B47] Keats MR Culos-Reed SN Courneya KS McBride M An examination of physical activity behaviors in a sample of adolescent cancer survivors J Pediatr Oncol Nurs 2006 23 3 135 142 10.1177/1043454206287304 16624889

[B48] Keats MR Culos-Reed SN A community-based physical activity program for adolescents with cancer (project TREK): program feasibility and preliminary findings J Pediatr Hematol Oncol 2008 30 4 272 280 10.1097/MPH.0b013e318162c476 18391695

[B49] Mostoufi-Moab S Ginsberg JP Bunin N Zemel BS Shults J Thayu M Leonard MB Body composition abnormalities in long-term survivors of pediatric hematopoietic stem cell transplantation J Pediatr 2012 160 1 122 128 10.1016/j.jpeds.2011.06.041 21839468 PMC3218257

